# Correction to “Genetic variation in G72 correlates with brain activation in the right middle temporal gyrus in a verbal fluency task in healthy individuals”

**DOI:** 10.1002/hbm.26756

**Published:** 2024-06-19

Krug A , Markov V , Krach S , Jansen A , Zerres K , Eggermann T , Stöcker T , Shah NJ , Nöthen MM , Georgi A , Strohmaier J , Rietschel M , Kircher T . Genetic variation in G72 correlates with brain activation in the right middle temporal gyrus in a verbal fluency task in healthy individuals. Hum Brain Mapp. 2011;1:118–126.10.1002/hbm.21005PMC687036120336655

A wrong table 2 had been submitted to the journal. The correct table 2 should read.

**Table:** TABLE II. Brain activation for the whole fMRI sample (*n* = 91), *P* < 0.05 corr., and cluster extent 20 voxels.

Region	Hem	BA	X	Y	Z	Ke	T
**Semantic verbal fluency > Reading aloud**							
Fronto‐temporal	R					3516	10.29
Supplementary motor area	R	21	24	−12	37		8.59
Pars triangularis	R	46	21	23	20		8.33
Fronto‐temporal	L					2403	8.69
Primary motor cortex	L	4	−25	−24	34		8.51
Posterior cingulate	L	23	−17	−41	19		7.73
Inferior frontal	L	45	−21	31	5	682	8.12
Thalamus	R	‐	3	−10	0	45	6.14
**Reading aloud > Semantic verbal fluency**							
Subgenual cingulate	R	25	3	13	−4	2238	15.27
Supramarginal gyrus	L	40	−60	−28	34	4178	15.05
Posterior cingulate	R	23	8	−41	36	11,396	13.65
Visual associative cortex	L	19	−55	−68	1	596	12.42
Visual associative cortex	R	19	59	−65	15	659	11.33
Superior frontal gyrus	R	8	30	36	44	28	10.7
Supramarginal gyrus	R	40	35	−37	44	50	9.85

Coordinates are listed in Talairach and Tournoux atlas space [Talairach and Tournoux, 1988]. BA is the Brodmann area nearest to the coordinate and should be considered approximate.

We apologize for this error.

